# Environmental interactions between people and birds in semiarid lands of the Zapotitlán Valley, Central Mexico

**DOI:** 10.1186/s13002-020-00385-1

**Published:** 2020-06-05

**Authors:** Yessica Angélica Romero-Bautista, Ana Isabel Moreno-Calles, Fernando Alvarado-Ramos, Maurino Reyes Castillo, Alejandro Casas

**Affiliations:** 1 Escuela Nacional de Estudios Superiores Unidad Morelia (ENES Morelia), Universidad Nacional Autónoma de México, UNAM, Campus Morelia, Antigua Carretera a Pátzcuaro No. 8701, Col. Ex-Hacienda de San José de la Huerta, 58190 Morelia, MICH México; 2 Jardín Botánico Helia Bravo Hollis, Comunidad de Zapotitlán Salinas, Puebla, México; 3 Instituto de Investigaciones en Ecosistemas y Sustentabilidad (IIES), Universidad Nacional Autónoma de México, UNAM, Campus Morelia, Antigua Carretera a Pátzcuaro No. 8701, Col. Ex-Hacienda de San José de la Huerta, 58190 Morelia, MICH México

**Keywords:** Biocultural diversity, Ethnozoology, Ethno-ornithology, Agroforestry, Management, Zapotitlán Salinas, Puebla

## Abstract

**Background:**

Birds have been among the most important element in lives of humans around the world, due to their presence and abundance in practically all ecosystems. Zapotitlán Salinas, a community of the Tehuacán Valley, has been a site of interest for studying ecology of bird communities, but no previous studies addressing the relationship between humans and birds have been conducted in the area. Based on their local knowledge, people of the area shape the use and conservation of local bird fauna diversity, which is being maintained or lost under the influence of factors like commerce, tourism, agriculture intensification or abandonment, public policies for conservation, environmental changes, among others. This study aims to analyze the patterns of interactions between humans and birds in a context of high biocultural diversity with a long history and facing the environmental and social challenges of semiarid areas.

**Methods:**

Ecological sampling for documenting bird species richness was conducted from November 2015 to May 2017 in three transects that included agroforestry systems, forests, and fallow agricultural land. The method of counting by fixed radius points at 16 points within the study systems was used. Thirty in-depth interviews were conducted with local people who own land in the study areas by random sampling and choosing experts of Zapotitlán Salinas, Puebla, a semiarid natural protected area and, since 2019, recognized as Mixed World Heritage. Some visitor guides of the Helia Bravo Hollis Botanical Garden who are member of the community and local people who dedicated part of their time to catching birds were considered as local experts. They have experience in identifying birds through bird watching and listening to bird songs, as well as their knowledge on behavior and habits of the bird species occurring in their locality. We in addition conducted free listing of bird species recognized by people and interviews on ecological aspects, forms of use, and management of birds using a photographic catalog as visual stimulus. The following aspects were addressed with local people: (i) the bird species of Zapotitlán recognized by them; (ii) the biological, ecological, and behavioral knowledge about these species; (iii) the description of practices of use and management of these animals; (iv) the perceived changes regarding presence and abundance of the wild birds recognized; and (v) the regulations of these practices and uses.

**Results:**

Through the ecological sampling, we identified 89 bird species, a number representing nearly 68% of all species reported for the Zapotitlán Valley. The species recorded belong to 61 genera, 26 families, and 11 orders. Local people interviewed recognized 62 morphospecies occurring in their territory, and designated them with 50 local names. The interactions of local people with birds and the knowledge related to habitats and habits varied according to people’s daily life activities and the ways of inter-generational transmission of knowledge. The interactions identified respond to several motivations. The most intense are those of utilitarian nature (three types of use are recognized: nutritional, medicinal, and ornamental), while other less notorious but equally relevant are those related to the awareness and conservation of biological diversity, and aesthetic appreciation of nature. Also relevant are those interactions shaped by the people’s worldview since some species are interpreted as climatic environmental predictors, amulets, or omens.

**Conclusions:**

To understand the various human-bird environmental interactions, it is necessary not only to address the utilitarian assessment that species have in a specific place but also those associated with cultural expressions and the connection between these aspects. Lifestyles, traditions, and beliefs model intangible forms of use, such as the interpretation of climate predictors. Ancient roles of birds in local culture are ongoing, and new demand of ornamental birds from cities influences catching activities but local and regional regulations have contributed to maintain them below a critical level.

## Background

Wildlife has been an important element in lives of humans around the world, and currently is a highly valued component of biocultural diversity. Such valuation is due to reasons of both utilitarian nature since it allows to cover basic needs, and cultural reasons since different animal species have had symbolic-religious attributes [1].

Among the faunal groups, human beings have been related to birds in various ways due to their presence in practically all ecosystems, their high abundance, and the ease of establishing contact with them [2]. In Mexico occur between 1123 and 1150 species [3, 4], which represent nearly 11% of the bird species richness existing in the world [5]. This country is currently the eleventh country with higher bird species richness and the fourth with higher endemism, with 194 to 212 species having some degree of endemism [5].

As a result of the interactions between human cultures and birds, these vertebrates have been used for multiple purposes; the most frequent being food and medicinal. This pattern reflects the needs of humans in the areas where birds are used and from this information it is possible to define the importance of wild, managed, or domesticated birds [6–8].

The capture and commercialization of birds for ornamental purposes is a pre-Hispanic practice that currently persists in various communities in Mexico [9]; the most popular species for this purpose being pigeons (*Zenaida asiatica* (Linnaeus, 1758); *Columbina inca* (Lesson, 1847)), the cenzontle (*Mimus polyglottos* (Linnaeus, 1758)), and several species of psittacids, such as parakeets (*Aratinga nana* (Vigors,1830); *Amazona albifrons* (Sparrman, 1788)); species of the Cardinalidae (cardinals, grosbeaks), Emberizidae (sparrows), Parulidae, and Corvidae [6, 10–12], which are marketed and maintained by households.

In rural communities, it is common to observe that a species can have more than one use and the parts of an individual can be destined to different purposes; also, people look for an integral use of bird species [6, 13].

In addition to their direct use, birds are associated to the announcement of rains, the description of changes in winds (climatic and environmental changes predictors), they are part of legends and omens of events, and they may be protecting as amulets [13–16]. Use of birds as omens and predictors are based on culturally learned and shared meanings in various human communities; this relationship makes birds dynamic elements in societies, beyond their ecological role in ecosystems [16].

It has been proposed that the richness of birds is directly related to the amount of rainfall in a region [17], but it is now also recognized that birds can be a diverse group in arid areas in correlation with the degree of environmental heterogeneity [18]. For instance, in the Tehuacán-Cuicatlán Biosphere Reserve (TCBR), bird fauna diversity is as high as that of areas dominated by tropical deciduous forests, and greater than that of other important arid areas of North America, even though the region has a smaller area [18, 19]. Such state of diversity is related with the high diversity of other biological groups and because of the ecosystem heterogeneity existing in that region [20].

Within this important semiarid zone, the village of Zapotitlán Salinas has been a site of special interest for the study of bird species richness, ecological interactions, and bird community dynamics [21]. However, no previous studies have been carried out addressing the relationship between humans and birds.

Zapotitlán has had relevant social-ecological changes in the last 20 years, which have influenced interactions between people and nature. Among these changes, the decree of the region as a Natural Protected Area (NPA) in 1998 had relevance [20]. As consequence, several communitarian regulations were constructed to have access to ecosystems and biodiversity [20, 22]. In addition, it should be mentioned social changes associated to migration of people and occupational changes from a main agriculturalist way of life to a tourism practice [22–24], as well as environmental changes such as marked interannual climatic variations, as recorded by local and regional climatic stations and other environmental changes, mainly changes in vegetation cover or its protection [21, 25, 26]. In such context, the main objective of this study was to analyze the environmental interactions between local people and birds in this town in relation to the social and ecological changes occurring in the area. We documented (i) the local environmental knowledge that the inhabitants have about the birds in their community, (ii) the use and management practices of birds and possible changes over time, and (iii) the existing rules and agreements in relation to the management of the birdlife.

## Methods

### Study area

**Fig. 1:**
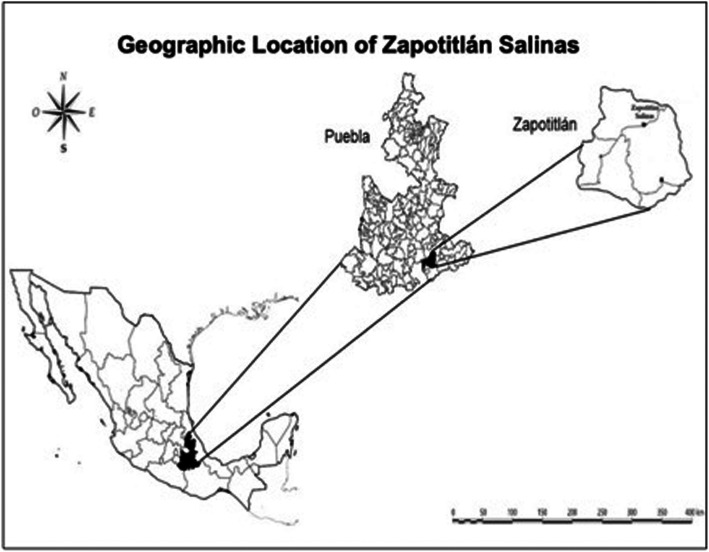
Location of Zapotitlán Salinas, Puebla in the Tehuacán-Cuicatlán Biosphere Reserve, Central Mexico

**Fig. 2:**
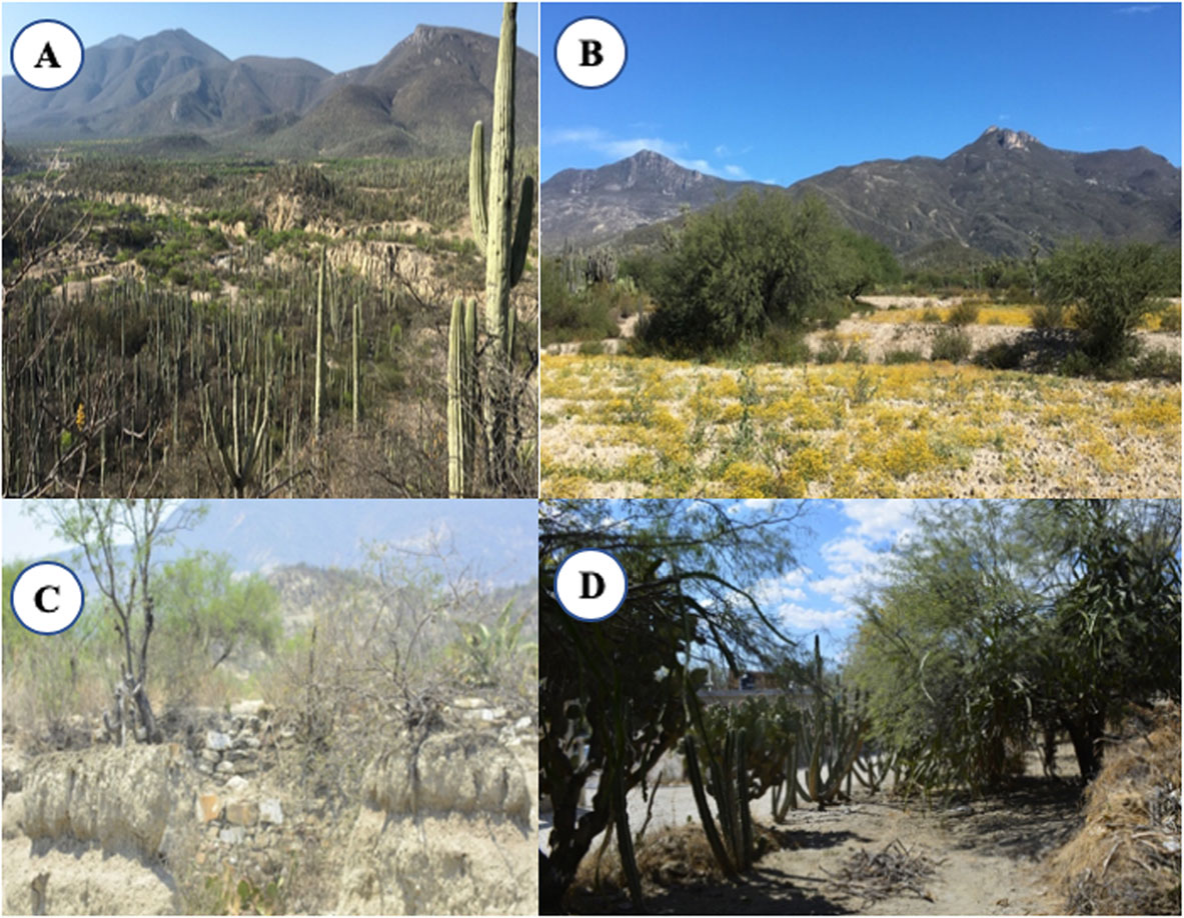
**a** Landscape of Zapotitlán Salinas vegetation from Cerro Cutá. **b** Agroforestry system of semi-terraces (cuaxustles). **c** Semi-terraces in a plot. **d** Homegarden within the town of Zapotitlán Salinas

Zapotitlán Salinas is the municipality located in western TCBR with a dry, semi-warm climate scanty rainy season in summer, with annual average of rainfall and temperature of 425 mm and 21.2 °C, respectively [27] (Fig. 1). Three territorial assemblages are identified: the communal land, the municipal head urban area, and the Botanical Garden Helia Bravo Hollis, which is formed by 100 ha exclusively dedicated to conservation [28]. The vegetation types occurring in this area are thorns-crub, tropical deciduous forest and xerophilous forest, and pastureland, with chaparral or Mexical in the hills surrounding the valley [29], and the vegetation communities are *mezquital*, thorn forest, columnar cacti forests (locally called *tetechera* and *cardonal*), the rosetophyllous forest *izotal*, the *tetechera-candelillar*, and the tropical dry forest [29–32] (Fig. 2). Regarding the herpetofauna of Zapotitlán Salinas, seven and 33 species of amphibians and reptiles have been reported, respectively [33], and 131 mammal species reported for the TCBR [34]. There is a list of insects still under construction, which includes edible thumbtacks, maguey worms, butterfly larvae, ants, bees, scorpions, among others [35, 36].

A total of 130 species of birds have been registered in the study area, belonging to 13 orders and 36 families, 18 of which are considered endemic to Mexico and 14 under some protection category in the NOM-059 or according to IUCN [21].

Currently, Zapotitlán Salinas is an attractive area for tourism, and part of people live from this activity as tourist guides, handcraft makers, and sellers, among others. But most people are dedicated to subsistence agriculture, free grazing of goats, salt production in mines, and harvesting wild resources [37, 38]. The decree of the region as an NPA, together with the inhabitants’ perception of water scarcity in the last 15 years, and changes in the forms of organization, have been detrimental for agricultural-livestock activities, giving way to the gradual abandonment of milpa-based agriculture, the increase in cash crops, and the increasing of activities related to tourism and offer of services [22, 39]. Only about 5% of the total population of Zapotitlán Salinas continues cultivating land annually [22].

### Research design

#### Sampling of bird species richness in agroforestry systems

##### Method for 50 m fixed radio counting points

Through this method, the observer remains at each fixed point and records all the birds observed and heard in an established area, for 10 min; recording in a format the counting point, the time of observation, the species, as well as age and sex of the individual recorded [40].

A total of 16 counting points was distributed in three transects, located in Paraje de la Monja (eight points), El Chichipe (four points), and San Martín (four points). The transects were placed in areas where there are agroforestry fields, semi-terraced areas with active agricultural use, or fallow agricultural areas and forest. This decision was taken because people commented that they observe or interact more frequently with birds in these management systems, in addition to other sites, mainly forests and homegardens.

Sampling was carried out from November 2015 to May 2017 (Table 1), covering different seasons throughout the year to observe patterns and changes along the different periods during the migratory season (in November) and the nesting season (from April to August), according to the information reported by Arizmendi and Espinosa de los Monteros [19].

**Table 1:** Bird sampling with the method of 50 m fixed radio count points

Sampling date	Sampling area	Number of points	Number of repetitions
November 2015	La Monja	Eight points	Sampled for 2 days
July 2016	La Monja	Eight points	Sampled for 2 days
October 2016	La Monja El Chichipe San Martín	Eight points Four points Four points	Sampled for 4 days Sampled for 4 days Sampled for 4 days
May 2016	La Monja El Chichipe San Martín	Eight points Four points Four points	Sampled for 4 days Sampled for 4 days Sampled for 4 days

The sampling was carried out in this way because the last two places were chosen later, selecting characteristics like the first: presence of the semi-terrace system, surrounded by the cacti forest; this in order to achieve a representative sampling at landscape scale

#### Interviews

Thirty semi-structured interviews [41] were conducted, from January to September 2017, with the purpose of describing the practices, beliefs, norms, institutions, and local environmental knowledge on birdlife. At the beginning of all the interviews, a free listing of the birds that the interviewed person knew was carried out.

During the interviews, the following aspects were addressed: (i) Zapotitlán birds that are recognized by local people; (ii) the biological, ecological, and behavioral knowledge that people have about the mentioned species; (iii) the existence and description of management practices and ways of using birds; (iv) the perceived changes and causes of these changes in both abundance and access to wild birds as resources; and (v) the governance (norms, agreements, and institutions) that exist in relation to people and birds interactions.

Out of 30 interviews conducted, 23 were carried out to one person and seven to two persons of a household simultaneously, in total 37 persons, 22 of them women and 15 men between 32 and 91 years old. The interviews were conducted with farmers who own land, homegardens owners, and local experts. We considered as local experts those tourist guides of the Helia Bravo Hollis Botanical Garden and people who are or were dedicated to catching birds for sale or as ornament. We consider that there is a strong interaction between these people and birds, when developing their productive activities and daily activities in these places which are inhabited and visited by birds.

A stratified “snowball” sampling was used to select people to be interviewed, which consisted in choosing individuals from one or several key informants, who guided the interviewer to other people who might have relevant information [42]. Likewise, the number of respondents was determined based on the theoretical saturation model [43].

#### Photographic catalog

A photographic catalog of the bird species of the Zapotitlán Valley was presented to the interviewees as visual stimuli for the description of the local knowledge that is possessed about them [44]; this was elaborated from the inventory of species conducted, and images obtained from the Image Bank of the National Commission for the Knowledge and Use of Biodiversity in 2016 (CONABIO) [45].

The catalog was used for complementing the interviews, as an incentive to know details that could have been omitted during the answer to the specific questions; together with the interview, it allowed estimating the number of species that people know and identify.

### Data analysis

The interviews were fully transcribed for later analysis by using the Atlas.ti software [46], a program based on the grounded theory of Glaser and Strauss [47]; with tools for structuring and qualitative analysis. From the identification of main concepts and ideas, an arrangement of the information was made in units of analysis, then a categorization of all the documents was made and networks were created in the software. Based on this information, we performed the analysis and interpretation of what was recorded in the field with the construction of an explanatory narrative [43, 47].

The free listing of birds was analyzed using a cognitive salience index, the Sutrop index, which combines the frequency and the order of mention of a bird [48]. The index was calculated from the following formula: *S* = *F* / (*N*mP); where *F* is the frequency of mention of each bird, *N* is the total number of respondents, and mP the average position of mention of bird [48]. Calculations were performed with the Flares program (Free list analysis under R environment using Shiny).

## Results and discussion

### Bird richness in managed systems

We recorded 89 bird species, 68% of the 130 species that have been reported for the Zapotitlán Salinas Valley. The species recorded belong to 61 genera, 26 families, and 11 orders: highlighting the Parulidae (13 species), Tyrannidae (12 species), and Trochilidae, Troglodytidae, and Passerellidae (with seven species each). Twenty-one species (23%) have some degree of endemism, seven being strictly endemic to Mexico, 11 semi-endemic, and three quasi-endemics (see Additional file 1).

Four species are under some protection category of the NOM-059; three are subject to special protection (*Parabuteo unicinctus* (Temminck, 1824); *Falco peregrinus* Tunstall, 1771; *Aimophila notosticta* (P. L. Sclater & Salvin, 1868)) and one is considered threatened (*Geothlypis tolmiei* (J. K. Townsend, 1839)). In total, 59 species are resident and 30 migratory; 43 are insectivorous, 14 omnivorous, 13 granivorous, seven nectarivores, seven carnivorous, and five frugivorous.

Nearly 66% of the registered species are resident; they have a wide distribution within the locality, are generalist species, and are associated with areas of higher temperature and lower humidity, compared to the migratory species that reach Zapotitlán Salinas, like the clay-colored finch, the peregrine falcon, or the ruby-throated hummingbird [18, 49, 50].

A large percentage of the migratory species registered for the locality (62%) were observed during the sampling. These species are associated to areas with higher humidity conditions in the locality, like riparian zones of the Salado river, and their distribution is smaller [18, 49].

Regarding the endemic species occurring in the Zapotitlán Salinas Valley, all of them were recorded during the sampling; these have a wide distribution within the locality [50].

The species that were not registered are those associated with riparian areas and temporary and permanent rivers and streams; also, those found in restricted areas of the locality, specifically, in the riparian mesquite areas [49]. In addition to the species with restricted distribution, it is likely that, even if the sampling effort can be considered robust, some species considered rare or not abundant in the area were not detected in samples.

Although the resident species occurring in the sampled plots move throughout the Zapotitlán Valley, the presence of a high percentage of migratory species indicates that the area with agroforestry management systems may represent spaces of important resources seasonally used, even when the humidity conditions are less favorable than in other areas.

Although the agroforestry practices established in the sampling plots are not currently subject to intense management, their presence and distribution within the field and their composition may be defining the richness of the birdlife. It has been described that agroforestry systems can provide important resources for migratory birds, such as shelter, food, and facilitate the movement between patches [51].

According to Zuria and Gates [52], the size and complexity of agroforestry practices of the “land margin,” such as living fences, windbreak barriers, terraces, or hedges, as well as its proximity with patches or remnants of the original vegetation, determine the richness and composition of the bird community; as well as the characteristics that appear in the sampled sites and their associated agroforestry practices in Zapotitlán Salinas.

Likewise, the presence and abundance of mesquite (*Prosopis laevigata* (Willd.) M.C. Johnst., Fabaceae) is positively related to the richness of birds, due to the multiple resources offered by this species. Zuria and Gates [52] documented the presence of 61 species of birds, 36 resident, and 24 migratory in agricultural plots within agroforestry practices called “land margin” in an arid area of Guanajuato.

In addition, the sites have a high abundance of mesquite, but unlike the study of Zuria and Gates, in Zapotitlán Salinas, besides mesquite, other plant species of importance for birdlife were recorded, including several species of columnar cacti (*Myrtillocactus geometrizans* (Mart. Ex Pfeiff.) Console., Cactaceae; *Stenocereus stellatus* (Pfeiff.) Riccob., Cactaceae), agaves (*Agave salmiana* Otto ex Salm-Dyck, Asparagaceae; *Agave marmorata* Roezl, Asparagaceae), legume (*Vachellia constricta* (Benth.) Seigler & Ebinger, Fabaceae; *Prosopis laevigata*), *Bursera* trees (*Bursera aptera* Ramirez, Burseraceae; *Bursera submoniliformis* Engl, Burseraceae), and bushes (*Jatropha neopauciflora* Pax, Euphorbiaceae, *Cnidoscolus* sp., Euphorbiaceae), among others.

### What people know about birds

Local people of Zapotitlán Salinas continually interact with birds of their locality; in homegardens, in the squares and streets of the town, as well as in the cultivated and fallow agricultural areas, and in the secondary and primary forest areas. According to the daily life and productive activities that each villager performs, these interactions may differ in diversity, and the groups of birds that are more commonly used and managed. From these interactions, the local people of Zapotitlán interviewed recognized 62 morphospecies occurring in their territory, which belong to 26 families and 11 orders. The difference with respect the general inventory generated during the research can be explained because for local people several species of the same order or genus are not differentiated. Most birds have a local name, which may be related to bird characteristics like color and shape of the feathers, their song, movement, feeding habits, specific behaviors (during foraging or courtship, for example) among other ecological habits [53, 54]. Villagers assign 50 local names to the 62 recognized morphospecies, because some species have different names, such as *Pyrocephalus rubinus* (Boddaert, 1783), which is known as “rayito,” or “San Gabrielito.” Contrarily, several species of the same genus have the same name, such as *Catherpes mexicanus* (Swainson, 1829), *Troglodytes aedon* Vieillot, 1809, and *Thryomanes bewickii* (Audubon, 1827), which are all called “saltapared.” People also recognize some species as varieties, such as orioles, three types of which are identified: “common oriole” (*Icterus wagleri* P. L. Sclater, 1857), “yellow oriole” (*Icterus pustulatus* (Wagler, 1829)), and “fine or Spanish oriole” (*Icterus cucullatus* Swainson, 1827) (see Additional file 2).

The recognized species are mostly residents and considered common in the locality, unlike most of the non-annealed species, which have a smaller distribution, associated with areas of higher humidity and lower temperature, that are considered rare or less abundant, like the migratory chipe *Mniotilta varia* (Linnaeus, 1766), or the few abundant *Sporophila torqueola* (Bonaparte, 1850)*.*

From the free listing of birds and the calculated cognitive salience index, six of the total birds mentioned are outstanding: *Mimus polyglottos* (Linnaeus, 1758) (0.33), *Zenaida asiatica* (Linnaeus, 1758) (0.24), *Haemorhous mexicanus* (P. L. Statius Müller, 1776) (0.19), *Icterus* sp. (0.15), *Columbina inca* (Lesson, 1847) (0.12), and *Toxostoma curvirostre* (Swainson, 1827) (0.11). The six species highlighted according to the order and frequency of mention are valued in several aspects; their use is an important aspect, since the six species are used as ornamental birds. *Columbina inca* and *Zenaida asiatica* are consumed as food, but also for being interpreted as signs, because *Toxostoma curvirostre* is considered a bird predictor. It is important to highlight that *Mimus polyglottos* was mentioned by all the interviewees and the highest index of valuation; it is important for its use, symbolic and aesthetic reasons.

### Interactions shaped by uses, beliefs, and rituals

The environmental interactions between people and birds of Zapotitlán Salinas respond to various motivations. Those more intense are utilitarian, while other less obvious are those related to the awareness of risk and conservation of biodiversity, as well as to the aesthetic appreciation and interactions shaped by the worldview of the population and interpreted from the knowledge they possess.

Eighteen species and the Trochilidae were registered with use, under three categories: food (four species), medicinal (four species and one family), and use for ornament (13 species). These species belong to ten families and six orders; within the Trochilidae, there is no selection by species for use due to the difficulty of selecting the individual when hunting (Table 2).

**Table 2:** Uses of the birds in Zapotitlán Salinas, Puebla, Mexico

Local Name	Use	Description
Tortolita (*Columbina inca*)	Food	It is consumed roasted or “barbecue” type; it can be accompanied with rice
Torito (*Columbina passerina*)	Food	It is consumed roasted
Carpintero (*Melanerpes hypopolius*)	Food	Its consumption is less common; it is also prepared roasted
Paloma tehuacanera, Torcasa (*Zenaida asiatica*)	Food	It is consumed roasted
Zopilotes (*Coragyps atratus*, *Cathartes aura*)	Medicinal	Used to cure rabies, through the use of their blood/used as a cancer treatment; the meat is consumed in a soup
Cacalote (*Corvus corax*)	Medicinal	Used to cure rabies and to treat “mishcahue.” It is prepared boiled together with palo blanco and casahuate (*Ipomoea* sp.)
Correcaminos (*Geococcyx velox*)	Medicinal	Used to treat epileptic seizures, heart diseases, and as a cancer treatment. The meat is consumed
Chuparosas, Chupamirtos (Trochilidae)	Medicinal	1. Used to treat “alferecia” and “tiricia.” It is plucked and boiled; the broth is consumed, blood is also used, and some consume the meat 2. Used to treat heart diseases. The whole bird is used and placed as a plasto in the children's chest The blood and boiled meat of the animal is ingested The animal is placed in alcohol and taken in small doses or spread. In the case of children, it is ingested prepared in tea 3. Also used to treat “mischcahue.” The animal is put in a jar with basil, ruda or other air plants and the liquid is ingested
Tortolita (*Columbina inca*)	Ornament	It is appreciated for its song
Torito (*Columbina passerina*)	Ornament	It is appreciated for its song
Gorrión rojo (*Haemorhous mexicanus*)	Ornament	It is appreciated for the color of its plumage and its song
Calandrias (*Icterus cucullatus*, *Icterus pustulatus*)	Ornament	Both species are appreciated for the color of their plumage
Chape (*Mimus polyglottos*)	Ornament	The cenzontle is appreciated as an ornamental bird for its many songs and ability to learn and imitate
Bionches (*Pheucticus chrysopeplus, Pheucticus melanocephalus*)	Ornament	Both species are appreciated for the color of their plumage
Dominico (*Spinus psaltria*)	Ornament	It is appreciated for the color of its plumage
Cuicuite (*Toxostoma curvirostre*)	Ornament	It is appreciated for its song
Primavera (*Turdus migratorius*)	Ornament	It is appreciated for its singing and the color of its plumage
Paloma tehuacanera, Torcasa (*Zenaida asiatica*)	Ornament	It is appreciated for its singing and the color of its plumage

*ECP* environmental and climatic changes predictors

**Fig. 3:**
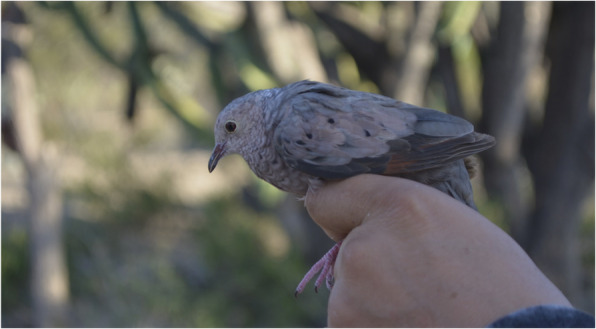
*Columbina passerina*, known as “torito” in Zapotitlán Salinas, Puebla, where it is used for food purposes

Three species of columbiforms (*Columbina inca*, *Columbina passerina* (Linnaeus, 1758) and *Zenaida asiatica*) were mentioned as the most common birds for consumption (Fig. 3), in addition to a species of woodpecker (*Melanerpes hypopolius* (Wagler, 1829)), which was mentioned less frequently, due to the difficulty of catching it.

For a medicinal use, the two species of vulture of the locality (*Coragyps atratus* (Bechstein, 1793) and *Cathartes aura* (Linnaeus, 1758)), the crow (*Corvus corax* Linnaeus, 1758), the roadrunner (*Geococcyx velox* (Wagner, 1836)), and several species of hummingbirds (Trochilidae) are used. The diseases that are treated with these species are mainly cardiac illnesses, epilepsy, and rabies; these birds are also used to treat conditions and discomforts such as “tirisia,” “alferecia,” and “mishcahue,” which are cultural diseases common in Zapotitlán Salinas. “Alferecia” is a condition of the children manifesting bruising of nails, lips, and eyelids, as well as seizures. “Mishcahue” is the upset that women feel after labor if they do not meet the recommended 40 days of resting, while “tirisia” is a condition whose symptoms are associated with lack of appetite, reluctance, and paleness; it usually occurs in people who suffer from sadness and disappointment [55].

Thirteen species were recorded with ornamental use: mainly the “chape” (*Mimus polyglottos*), the “tehuacanera dove” (*Zenaida asiatica*), the tortolita (*Columbina inca*), the “torito” (*Columbina passerina*), the sparrow (*Haemorhous mexicanus*), two species of orioles (*Icterus cucullatus*, *Icterus pustulatus*), two species of “bionches” (*Pheucticus chrysopeplus* (Vigors, 1832), *Pheucticus melanocephalus* (Swainson, 1827)), “cuicuite” (*Toxostoma curvirostre*), and “dominico” (*Spinus psaltria* (Say, 1822)). Other species that come to be kept at home, but less frequently, are the nightingale (*Phainopepla nitens* (Swainson, 1838)) and “primavera” (*Turdus migratorius* Linnaeus, 1766).

Edible and medicinal uses of birds have declined, mainly because, unlike in the past, residents can now access to other resources and services, such as commercial meat and a nearby health service. Currently, food use is linked to childhood activities, while medicinal use is even more restricted to those who have the knowledge, usually to attend emergencies or as an alternative treatment to the one they receive from the physician.

While the maintenance of local ornamental birds is a practice, which has decreased but persists, since it is considered that “birds bring joy to the house” and various interactions with them are established.

The cenzontle, the sparrow, that one also sings a lot, he learns, he also learns if you teach him. The sparrow, the bionche, they do learn, usually the people have sparrows, almost in the house they have the sparrow, the cenzontle; they are attractive because the sing”—Woman, 84 years.

Other forms of interactions with birds were recorded in the site studied, linked to beliefs, rites, and rituals and to the reading or predicting environmental events. The use as amulet was registered in a family, which represents an element of great value for the person who carries it and transmits and secures a good. We recorded six species as omens, which are signs of warning of a future event. The category of climatic and environmental changes predictors (ECP nine species) is understood as the reading and interpretation of a signal expressed from an unusual song or activity of the bird, which indicates a change in the weather or a specific climatic event and finally, the ritual category (a species and a family) is registered, in which a bird is used as an element associated with an event of ritual characteristics (Table 3).

**Table 3:** Bird species with categories linked to beliefs, myths, rites, and rituals in Zapotitlán Salinas

Local Name	Category	Description
Chuparosas, Chupamirtos (Trochilidae)	Amulet	It is used as an amulet for good luck. It is captured and dried to be placed on the door of the home or loaded in the bag.
Zopilotes (*Cathartes aura, Coragyps atratus*)	Omen	Announces bad luck when it crosses the person’s path.
Correcaminos (*Geococcyx velox*)	Omen	Announces bad luck when it crosses the person’s path.
Tecolote (*Glaucidium brasilianum*)	Omen	Announces the death of a relative by singing at night, insistently, on the side of the house.
Rayito, Pájaro del rayo, San Gabrielito (*Pyrocephalus rubinus*)	Omen	Announces good luck when the person sees one.
Cuicuite (*Toxostoma curvirostre*)	Omen	Announces a visit from a family member when it sings insistently near the house.
Totopito con chilaquil (*Aegolius acadicus*)	ECP	He announces cold or rain with his insistent song.
Correcaminos (*Geococcyx velox*)	ECP	It announces rain and strong wind with its song, which is rare because it is a bird that does not sing frequently. Announces heat through a different song, which is interpreted as cheerful.
Tecolote (*Glaucidium brasilianum*)	ECP	Announces the beginning of the rainy season, or enough rain during the season with its song during the early morning.
Golondrina (*Stelgidopteryx serripennis*, *Hirundo rustica*)	ECP	It indicates nearby rain when rising at high altitude during its flight in open places.
Saltapared (*Catherpes mexicanus*, *Troglodytes aedon*, *Thryomanes bewickii*)	ECP	Announces the rain with his insistent song.
Lechuza (*Tyto alba*)	ECP	Announces the cold with his song.
Cacalote (*Corvus corax*)	Ritual	Blood is used as an element in a ritual against witchcraft.
Chuparosas, Chupamirtos (Trochilidae)	Ritual	It is used as an element to make clean against “mal de ojo” or “aire”

The Trochilidae is used as amulet; no any particular species of hummingbird is preferred; their capture is carried out opportunistically or casually, and the hummingbird captured can be kept for later use as amulet or medicine.

**Fig. 4:**
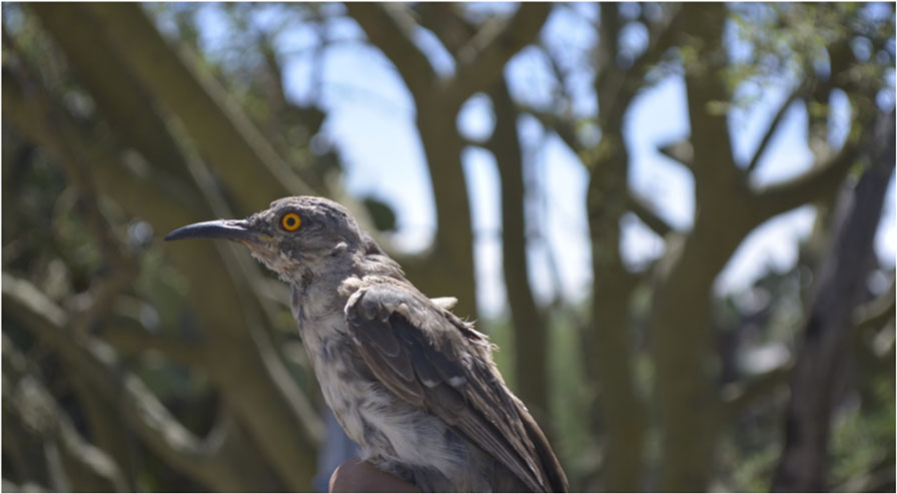
*Toxostoma curvirostre,* named “cuicuite” in Zapotitlán Salinas, is a bird that warns with its song about upcoming visits

Used for omen, we recorded the two species of vultures, the roadrunner, the “tecolote” (*Glaucidium brasilianum* (Gmelin, 1788)), the “rayito,” and the “cuicuite” (Fig. 4). These are divided between those that presage or announce good luck or an event considered good and those that presage bad luck or notify about an event considered bad.

Nine species were recorded as predictors of climatic and environmental changes: the “totopito con chilaquil” (*Aegolius acadicus* (Gmelin, 1788)), the roadrunner, the “tecolote,” two species of swallows (*Hirundo rustica* Linnaeus, 1758*; Stelgidopteryx serripennis* (Audubon, 1838)), three species recognized as “saltapared” (*Catherpes mexicanus* (Swainson, 1829); T*roglodytes aedon* Vieillot, 1809; *Thryomanes bewickii* (Audubon, 1827)), and the owl (*Tyto alba* (Scopoli, 1769)). In the case of peasants and salineros (people extracting salt from mines), these ECPs help them to interpret rain announcements that allow them to prepare actions; for instance, the salineros rush the collection and protection of salt in the face of this rain forecast.

Also, the one who sings is the saltapared, sings also when is going to rain. Ah! also the roadrunner warns, that almost does not sing, that, when it is very hot and the time is already set then he sings and it is going to rain, yes—Man, 73 years.

Finally, *Corvus corax* and species of the Trochilidae were registered in the ritual category, which are birds used as elements within rituals of limpias (spiritual cleaning).

### Management practices

Three practices of bird management were identified: hunting, capture, and bird nurturing. The birds that are under these practices are subsequently destined to various forms of use.

#### Hunting

Bird hunting is carried out only with a slingshot and is intended to obtain individuals for food use, medicinal use, or to prepare and preserve the bird as amulet. This activity is performed in hills or agricultural fields occasionally; in the case of food use, hunting is done for immediate use, while birds hunted for medicinal use or as amulet can be kept for later use (Tables 2 and 3).

#### Capture

**Fig. 5:**
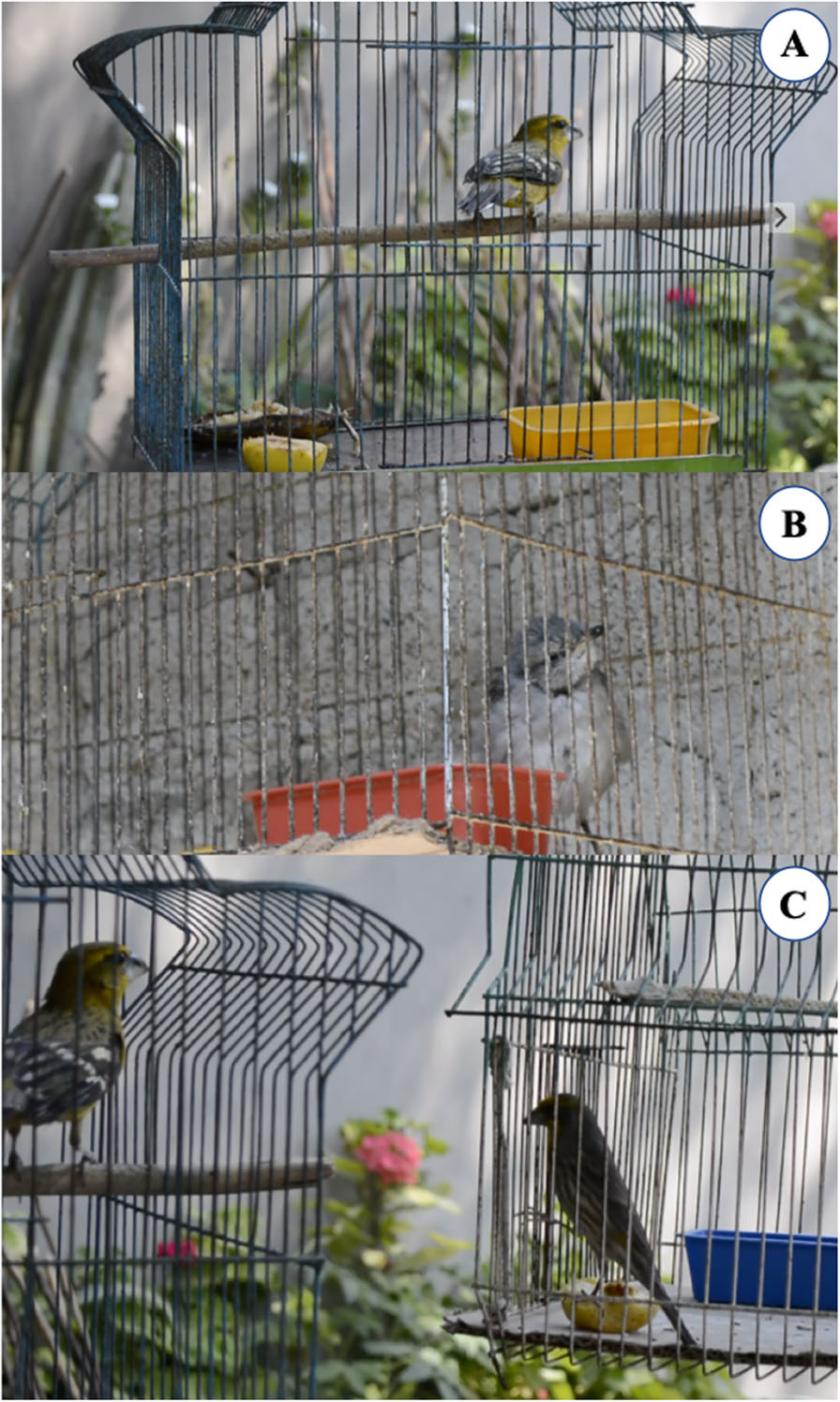
Ornamental birds in Zapotitlán Salinas. **a***Pheucticus chrysopeplus* (bionche)*.***b***Mimus polyglottos* (chape). **c***Haemorhous mexicanus* (gorrión)

The chicks of several bird species are captured in the nest, occasionally, while other activities are carried out in hills or plots. Such are the cases of grazing and caring for cattle or goats, the preparation of the land, planting or harvesting of crops, harvesting of fruits, and inflorescences of cacti or insects, can also be carried out by a specific order; the birds that are caught are destined only as ornamental birds (Fig. 5).

In general, children and women make the capture from April to August. Some men also involve in the capture and take the chicks to their homes. Occasional captures are also made in the homegardens; in addition, it is common that people give a bird as present.

In the forest and agricultural fields, nests are identified through the behavior of adult individuals; once located, one or all the chicks are captured, depending on the amount of chicks found in the nest, and according to the species. Although no specific sites for capturing chicks were identified, the canyons, cacti forest, and the agroforestry systems were referred to as the more frequented places for carrying out this practice.

The male individuals are selected for captures since these have more intense coloring of the plumage than females and emit striking songs; both characteristics are appreciated in ornamental birds. Selection of male chicks is carried out according to their size; the largest chicks being males, and the “transparency” of the beak; according to the handlers, the nostrils of the chicks should be observed; if it can be seen through the holes in the chick, the chick is most likely male. People decide to leave the females in the nest so that these species continue to reproduce.

### Bird nurturing

In Zapotitlán Salinas, there has not been documented reproduction of wild birds in captivity. Whether the chicks are destined to be kept in the captor’s house or is intended for sale, it must be taught to eat; it is fed with a variety of native cactus fruits, in addition to other domesticated fruits, seeds, and other foods such as dough, chickpea, chili, goat milk, and boiled egg, depending on the species.

In addition, birds must get used to people and the noise that surrounds the house so to ensure their survival; they cannot be sold until that period of adaptation ends and eats by themselves; this process is carried out mostly by women or children guided and helped by adults.

### Persistence and loss of forms of use and other categories of interaction

The use of birds for food and medicine are widespread throughout the country; several species of the Columbidae are selected for consumption; this preference may be related to the flavor of the meat, provided by its granivorous diet, with its presence and abundance in populated areas and with its hunting facility [7, 44, 56, 57].

The current medicinal use of hummingbird and vulture species in Mexico is related to the beliefs that various regions of the country have preserved since pre-Hispanic times; these birds had a strong presence and deep meaning in the Mesoamerican worldview [58]. The hummingbird represented rebirth, vitality, sexuality, and since then, healing powers were attributed to treat epilepsy, so its current use to treat heart disease represents a permanence of knowledge and this reading and interpretation in the community worldview. While the vulture had the function in the world of cleaning the rotten, had access and contact with the underworld, and represented purification [58, 59].

### Norms, agreements, and transmission of local environmental knowledge

Management practices allow birds to continue to reproduce, ensure a greater probability of survival by avoiding the capture of adult individuals, and the capture of birds by people outside the community is monitored; when they identify someone with cages trying to catch birds, they attract their attention and prevent them from being carried away.

Local environmental knowledge linked to birds is transmitted generationally, from grandparents and parents to children and grandchildren. It is taught to recognize the birds and their songs and to identify the nests corresponding to the species, the migration seasons of the migratory species, the process of capture and nurturing in those families that perform this practice, as well as the reading and interpretation of the predictor birds or “poultry birds.”

This teaching-learning process is linked to experience and observation of the environment; it is transmitted while walking and working the field, through daily observation and the assessment, use and conservation of species.

### Persistence and loss of human-bird interactions and knowledge

Given the changes in the uses of the local birdlife, there is a process of loss of knowledge; knowledge linked to food and medicinal uses is more vulnerable to the passage of time, compared to those linked to reading the environment and cultural interpretations.

A hypothesis derived from this research is that this is related to the effort that needs to be invested to obtain the birds and use them directly, as well as with the persistence of the utilitarian value over time. In addition to the above, restrictions to using of wildlife in the community, as well as access to other resources, have led to the discontinuation of wildlife for nutritional and medicinal purposes.

On the other hand, for the interpretation of the ECPs and the omens, in which a reading of the environment and predictions is developed, these processes are still carried out to the extent that they are still useful for the daily life of the inhabitants. In addition, the teaching of these dynamics of reading and interpretation continue being transmitted, while observation allows the residents to establish complex relationships with their natural environment and from that generate environmental knowledge at the local level to deal with uncertainty in time and space [15].

### Environmental changes in Zapotitlán Salinas

The interviewees said that the raining patterns have changed, particularly the temporality and intensity of rains. Therefore, the capacity of predicting events of rain and their characteristics by people has decreased their effectivity, and there is a generalized perception of rainfall scarcity in the region.

Such perceived rainfall scarcity is the main explanation that people give to the decline of crop production in recent years, a situation that has led to a gradual abandonment of agricultural activities. Recently, alternative crops have been implemented (for example: pitahaya, papaya, agave); in addition, the increasing of tourism-associated activities and the provision of services (food services, lodging), as the main economic activities that the population consider more profitable than agriculture, has been increased.

It is also explained by people that such decrease in rainfall is the cause of changes in the structure in homegardens within the town, which have modified their composition, reducing the presence of plant species that require more water.

**Fig. 6:**
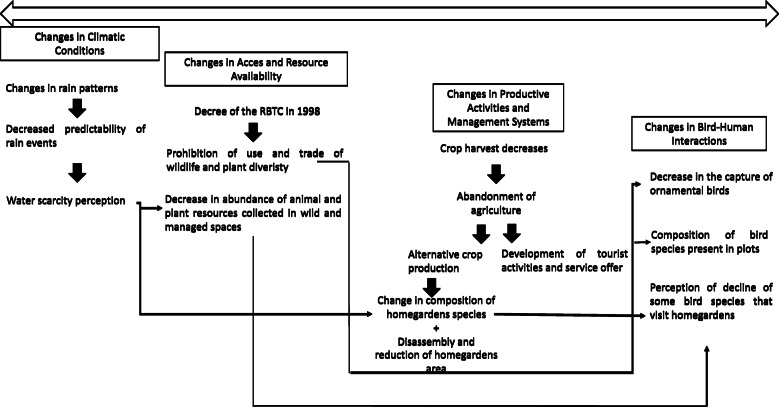
Environmental changes in Zapotitlán Salinas. It shows the events that at different temporal and spatial scales have modified interactions of people with birds are shown. The perceived shortage is the main explanation that people give to the decrease in crops in recent years, a situation that has led to a gradual abandonment of agriculture activities in Zapotitlán Salinas. It is also due to this scarcity of water, that other cultivation spaces, the homegardens within the town have been modified in terms of composition, reducing the presence of the species that require more water. Since the decree of the Tehuacán-Cuicatlán Biosphere Reserve, in 1998, the capture and sale of ornamental birds was prohibited; given these norms, the change in bird capture activities was gradual and has been reflected in the decrease in the sale of ornamental birds outside of Zapotitlán Salinas. Therefore, the people have noticed the increase of some species; in addition, the decrease of others is perceived, which the villagers relate to the decrease of some species of fruit trees in the homegardens and in the food availability in the forests and hills

The environmental changes that have taken place in Zapotitlán Salinas at different scales shape the relationship that people establish with their environment and with the biodiversity occurring in it. The interactions with the local birdlife have been transformed from the decree of the Reserve and other less obvious processes that have occurred gradually over time (Fig. 6). From the decree of the TCBR, in 1998, the capture and sale of ornamental birds was prohibited, as well as the use and trade of other faunal and plant groups. These prohibitions were stipulated in the management program of the Reserve.

Given these restrictions, the change in bird capture activities was gradual and has been reflected, to a greater extent, in the decrease of commercialization of ornamental birds outside Zapotitlán Salinas. Inside the town, the commercialization continues happening; however, this does not represent an important economic income for those who practice it, similarly as it happened in the past, according to local people perception.

Thus, we can see that other forms of interaction arose between local people and the birds of Zapotitlán, from the development of activities associated to tourism in the context of the TCBR. Bird watching, for instance, has emerged as an activity of interest to some visitors. In particular, it is attractive to foreign observers and to the academic sector that is dedicated to ornithological research, which can be facilitated by the local guides of the botanical garden, who have a detailed knowledge of its local bird diversity.

In addition to the time scale, there are defined spaces where the described interactions occur. The importance of the resources occurring in the semi-terrace plots (*cuaxustles*) and homegardens for the maintenance of the populations of some bird species is noticeable, since these may be functioning as spaces for resting and accessing food when they are scarce in the forest. The loss of some plant species in the homegardens, which can no longer be maintained due to the water availability, may be generating changes in these interactions; therefore, it seems important to conserve the homegardens that still maintain plant resources, both native and some introduced that do not require much water.

### Theoretical contribution from the environmental sciences perspective to approach human-bird interactions

Differently to other perspectives that focus on social or natural aspects, the ethnobiological perspectives make possible to identify the connection between use, management, and symbolism that human groups have in relation to birds and the representations that are made in a past and present temporal continuum [60]. Through investigating the local names that people give to their birds, we can know the diversity that a human group recognizes, identifying the characteristics that it observes of the species, as well as the cultural significance they possess.

The knowledge that we documented through this perspective includes the interpretation of birds as indicators of annual cycles and environmental quality, as a guide to some human actions, as well as elements of resource use regulation, which allows us to analyze its ecological and sociocultural importance, at various scales; since this knowledge although in principle is studied locally, it is possible then to find patterns at other scales [60, 61].

The environmental perspective is an approach that unifies social and ecological systems and contributes to ethnobiology allowing the approach to spatial and temporal scales in a dynamic way. Therefore, it is possible to link the ecological, economic, and social changes of the region to understand the impact of these factors in the human-bird relations beyond a static historical perspective.

## Conclusions

The great richness of birds of Zapotitlán, recognized and interpreted from the vast and detailed local environmental knowledge, is shown as a field of opportunity to develop eco-tourism-associated activities including bird watching and conservation. It is important to enhance the oral transmission of knowledge related to local diversity, through activities such as science popularization activities, which may contribute to prevent the loss and erosion of this knowledge.

To understand the various human-bird environmental interactions, it is necessary not only to address the utilitarian importance that species have in a specific place but also to address the importance of intangible cultural relationships and read the connection between these aspects, by resorting to it to the uses and to the system of beliefs and normativity that persist and resist. Ancient roles of birds in local culture are ongoing; the demand of ornamental birds from cities influences catching activities but local and regional regulations have contributed to maintain them below a critical level.

## Supplementary information

**Additional file 1: Table S1.** Species recorded in the samples. EN: endemic; SE: semiendemic; CE: quasiendemic; R: resident; MI: winter migratory; A: threatened; E: probably extinct in the wild; P: in danger of extinction; Pr: subject to special protection.

**Additional file 2: Table S2.** Species identified by the inhabitants and their local name. The local name is associated with: S: song; PC: plumage color; FH: feeding habits; RH: reproductive habits; BH: behavioral habits; FS: feather shape: M: movements; D: damage to the population.

## Data Availability

Data are attachment to the manuscript.
