# Supplementary material for: Environmental interactions between people and birds in semiarid lands of the Zapotitlán Valley, Central Mexico
Source: J Ethnobiol Ethnomed. 2020 Jun 5;16:32. doi: 10.1186/s13002-020-00385-1 (PMC7275383; doi:10.1186/s13002-020-00385-1)
Supplement: Supplementary file 1 — Additional file 1: Table S1. Species recorded in the samples. EN: endemic; SE: semiendemic; CE: quasiendemic; R: resident; MI: winter migratory; A: threatened; E: probably extinct in the wild; P: in danger of extinction; Pr: subject to special protection. [file 13002_2020_385_MOESM1_ESM.docx]

**Additional file 1**

1.Species recorded in the samples

| Order | Family | Species | Common name | Distri-bution | NOM-059 | Guild | Seasona-lity |
| --- | --- | --- | --- | --- | --- | --- | --- |
| Columbiformes | Columbidae | *Columbina inca* (Lesson, 1847) | Tortolita cola larga |  |  | Gran | R |
|  |  | *Columbina passerina* (Linnaeus, 1758) | Tortolita pico rojo |  |  | Gran | R |
|  |  | *Zenaida asiatica* (Linnaeus, 1758) | Paloma alas blancas |  |  | Gran | R |
|  |  | *Zenaida macroura* (Linnaeus, 1758) | Huilota común |  |  | Gran | R |
| Cuculiformes | Cuculidae | *Geococcyx velox* (Wagner, 1836) | Correcaminos tropical |  |  | Omn | R |
| Caprimulgiformes | Caprimulgidae | *Caprimulgus ridgwayi* (Nelson, 1897) |  |  |  | Inse | R |
| Apodiformes | Trochilidae | *Eugenes fulgens* (Swainson, 1827) | Colibrí magnífico |  |  | Nect | R |
|  |  | *Calothorax lucifer* (Swainson, 1827) | Colibrí Lucifer | SE |  | Nect | MI |
|  |  | *Archilochus colubris* (Linnaeus, 1758) | Colibrí garganta rubí |  |  | Nect | MI |
|  |  | *Cynanthus sordidus* (Gould, 1859) | Colibrí opaco | EN |  | Nect | R |
|  |  | *Cynanthus latirostris* Swainson, 1827 | Colibrí pico ancho | SE |  | Nect | R |
|  |  | *Amazilia beryllina* (Deppe, 1830) | Colibrí berilo |  |  | Nect | R |
|  |  | *Amazilia violiceps* (Gould, 1859) | Colibrí corona violeta | SE |  | Nect | R |
| Cathartiformes | Cathartidae | *Coragyps atratus* (Bechstein, 1793) | Zopilote común |  |  | Carn | R |
|  |  | *Cathartes aura* (Linnaeus, 1758) | Zopilote aura |  |  | Carn | R |
| Accipitriformes | Accipitridae | *Parabuteo unicinctus* (Temminck, 1824) | Aguililla rojinegra |  | Pr | Carn | R |
|  |  | *Buteo jamaicensis* (Gmelin, 1788) | Aguililla cola roja |  |  | Carn | R |
| Trogoniformes | Trogonidae | *Trogon elegans* Gould, 1834 | Coa elegante |  |  | Frug | R |
| Coraciiformes | Momotidae | *Momotus mexicanus* Swainson, 1827 | Momoto corona canela | CE |  | Inse | R |
| Piciformes | Picidae | *Melanerpes hypopolius* (Wagler, 1829) | Carpintero del Balsas | EN |  | Inse | R |
|  |  | *Melanerpes aurifrons* (Wagler, 1829) | Carpintero cheje |  | c | Inse | R |
|  |  | *Sphyrapicus varius* (Linnaeus, 1766) | Carpintero moteado |  |  | Inse | MI |
|  |  | *Picoides scalaris* (Wagler, 1829) | Carpintero mexicano |  |  | Frug | R |
|  |  | *Colaptes auratus* (Linnaeus, 1758) | Carpintero de pechera común |  |  | Inse | R |
| Falconiformes | Falconidae | *Falco sparverius* (Linnaeus, 1758) | Cernícalo americano |  |  | Carn | R |
|  |  | *Falco peregrinus* Tunstall, 1771 | Halcón peregrino |  | Pr | Carn | MI |
| Passeriformes | Tyrannidae | *Camptostoma imberbe* P. L. Sclater, 1857 | Mosquerito chillón |  |  | Inse | R |
|  |  | *Contopus virens* (Linnaeus, 1766) | Papamoscas del este |  |  | Inse | MI |
|  |  | *Empidonax occidentalis* Nelson, 1897 | Papamoscas amarillo barranqueño | SE |  | Inse | MI |
|  |  | *Sayornis saya* (Bonaparte, 1825) | Papamoscas llanero |  |  | Inse | MI |
|  |  | *Pyrocephalus rubinus* (Boddaert,1783) | Papamoscas cardenalito |  |  | Inse | R |
|  |  | *Myiarchus tuberculifer* (Orbigny & Lafresnaye, 1837) | Papamoscas triste |  |  | Inse | R |
|  |  | *Myiarchus cinerascens* (Lawrence, 1851) | Papamoscas cenizo |  |  | Inse | MI |
|  |  | *Tyrannus melancholicus* Vieillot, 1819 | Tirano pirirí |  |  | Inse | R |
|  |  | *Tyrannus vociferans* Swainson, 1826 | Tirano chibiú | SE |  | Inse | MI |
|  |  | *Tyrannus crassirostris* Swainson, 1826 | Tirano pico grueso | SE |  | Inse | MI |
|  |  | *Tyrannus verticalis* Say, 1823 | Tirano pálido |  |  | Inse | MI |
|  | Laniidae | *Lanius ludovicianus* Linnaeus, 1766 | Verdugo americano |  |  | Carn | R |
|  | Vireonidae | *Vireo griseus* (Boddaert, 1783) | Vireo ojos blancos |  |  | Inse | MI |
|  |  | *Vireo gilvus* (Vieillot, 1808) | Vireo gorjeador |  |  | Inse | MI |
|  | Corvidae | *Aphelocoma californica* (Vigors, 1839) | Chara de collar |  |  | Omni | R |
|  | Hirundinidae | *Stelgidopteryx serripennis* (Audubon, 1838) | Golondrina alas aserradas |  |  | Inse | R |
|  |  | *Hirundo rustica* Linnaeus, 1758 | Golondrina tijereta |  |  | Inse | R |
|  | Troglodytidae | *Salpinctes obsoletus* (Say, 1822) | Saltapared de rocas |  |  | Inse | R |
|  |  | *Catherpes mexicanus* (Swainson, 1829) | Saltapared barranqueño |  |  | Inse | R |
|  |  | *Troglodytes aedon* Vieillot, 1809 | Saltapared común |  |  | Inse | MI |
|  |  | *Thryomanes bewickii* (Audubon, 1827) | Saltapared cola larga |  |  | Inse | R |
|  |  | *Campylorhynchus jocosus* P. L. Sclater, 1860 | Matraca del Balsas | EN |  | Omni | R |
|  |  | *Campylorhynchus brunneicapillus* (Lafresnaye, 1835) | Matraca del desierto |  |  | Omni | R |
|  |  | *Pheugopedius maculipectus* (Lafresnaye, 1845) | Saltapared moteado |  |  | Inse | R |
|  | Polioptilidae | *Polioptila caerulea* (Linnaeus, 1766) | Perlita azul gris |  |  | Inse | R |
|  |  | *Polioptila albiloris* P. L. Sclater & Salvin, 1860 | Perlita pispirria |  |  | Inse | R |
|  | Regulidae | *Regulus calendula* (Linnaeus, 1766) | Reyezuelo matraquita |  |  | Inse | MI |
|  | Mimidae | *Toxostoma curvirostre* (Swainson, 1827) | Cuitlacoche pico curvo |  |  | Frug | R |
|  |  | *Mimus polyglottos* (Linnaeus, 1758) | Centzontle norteño |  |  | Omni | R |
|  | Ptiliogonatidae | *Phainopepla nitens* (Swainson, 1838) | Capulinero negro |  |  | Frug | R |
|  | Fringillidae | *Euphonia elegantissima* (Bonaparte, 1838) | Eufonia gorra azul |  |  | Frug | R |
|  |  | *Haemorhous mexicanus* (P. L. Statius Müller, 1776) | Pinzón mexicano |  |  | Omni | R |
|  |  | *Spinus psaltria* (Say, 1822) | Jilguerito dominico |  |  | Gran | R |
|  | Passerellidae | *Aimophila ruficeps* (Cassin, 1852) | Zacatonero corona canela |  |  | Gran | R |
|  |  | *Aimophila notosticta* (P. L. Sclater & Salvin, 1868) | Zacatonero oaxqueño | EN | Pr | Gran | R |
|  |  | *Melozone fusca* (Swainson, 1827) | Rascador viejita |  |  | Gran | R |
|  |  | *Melozone albicollis* (P. L. Sclater, 1858) | Rascador oaxaqueño | EN |  | Gran | R |
|  |  | *Peucaea humeralis* (Cabanis, 1851) | Zacatonero pecho negro | EN |  | Gran | R |
|  |  | *Peucaea mystacalis* (Hartlaub, 1852) | Zacatonero embridado | EN |  | Gran | R |
|  |  | *Spizella pallida* (Swainson, 1832) | Gorrión pálido | SE |  | Omni | MI |
|  | Icteridae | *Icterus wagleri* P. L. Sclater, 1857 | Calandria de Wagler |  |  | Omni | R |
|  |  | *Icterus spurius* (Linnaeus, 1766) | Calandria castaña |  |  | Inse | MI |
|  |  | *Icterus cucullatus* Swainson, 1827 | Calandria dorso negro menor | SE |  | Omni | MI |
|  |  | *Icterus pustulatus* (Wagler, 1829) | Calandria dorso rayado |  |  | Inse | R |
|  |  | *Molothrus aeneus* (Wagler, 1829) | Tordo ojos rojos |  |  | Gran | R |
|  | Parulidae | *Mniotilta varia* (Linnaeus, 1766) | Chipe trepador |  |  | Inse | MI |
|  |  | *Leiothlypis peregrina* (A. Wilson, 1811) | Chipe peregrino |  |  | Inse | MI |
|  |  | *Leiothlypis celata* (Say, 1822) | Chipe oliváceo |  |  | Inse | MI |
|  |  | *Leiothlypis ruficapilla* (A. Wilson, 1811) | Chipe cabeza gris |  |  | Inse | MI |
|  |  | *Leiothlypis virginiae* (S. F. Baird, 1860) | Chipe de Virginia | SE |  | Inse | MI |
|  |  | *Geothlypis tolmiei* (J. K. Townsend, 1839) | Chipe lores negros |  | A | Inse | MI |
|  |  | *Setophaga americana* (Linnaeus, 1758) | Chipe pecho manchado |  |  | Inse | MI |
|  |  | *Setophaga coronata* (Linnaeus, 1766) | Chipe rabadilla amarilla |  |  | Omni | MI |
|  |  | *Setophaga nigrescens* (J. K. Townsend, 1837) | Chipe negrogris | SE |  | Inse | MI |
|  |  | *Setophaga townsendi* (J. K. Townsend, 1837) | Chipe de Townsend |  |  | Inse | MI |
|  |  | *Setophaga virens* (J. F. Gmelin, 1789) | Chipe dorso verde |  |  | Inse | MI |
|  |  | *Basileuterus rufifrons* (Swainson, 1838) | Chipe gorra canela | CE |  | Inse | R |
|  |  | *Cardellina pusilla* (A. Wilson, 1811) | Chipe corona negra |  |  | Inse | MI |
|  | Cardinalidae | *Pheucticus chrysopeplus* (Vigors, 1832) | Picogordo amarillo | CE |  | Omni | R |
|  |  | *Pheucticus melanocephalus* (Swainson, 1827) | Picogordo tigrillo | SE |  | Omni | R |
|  |  | *Passerina caerulea* (Linnaeus, 1758) | Picogordo azul |  |  | Omni | R |
|  |  | *Passerina versicolor* (Bonaparte, 1838) | Colorín morado |  |  | Omni | R |
|  | Thraupidae | *Sporophila torqueola* (Bonaparte, 1850) | Semillero de collar |  |  | Gran | R |

**EN:** endemic; **SE:** semiendemic; **CE:** quasiendemic; **R:** resident; **MI:** winter migratory

**A:** threatened; **E:** probably extinct in the wild; **P:** in danger of extinction; **Pr:** subject to special protection
