# Supplementary material for: Environmental interactions between people and birds in semiarid lands of the Zapotitlán Valley, Central Mexico
Source: J Ethnobiol Ethnomed. 2020 Jun 5;16:32. doi: 10.1186/s13002-020-00385-1 (PMC7275383; doi:10.1186/s13002-020-00385-1)
Supplement: Supplementary file 2 — Additional file 2: Table S2. Species identified by the inhabitants and their local name. The local name is associated with: S: song; PC: plumage color; FH: feeding habits; RH: reproductive habits; BH: behavioral habits; FS: feather shape: M: movements; D: damage to the population. [file 13002_2020_385_MOESM2_ESM.docx]

**Additional file 2**

2. Species identified by the inhabitants and their local name

| Order | Family | Species | Local name | Local name annotations |
| --- | --- | --- | --- | --- |
| Columbiformes | Columbidae | *Columba livia* | Pichón |  |
|  |  | *Columbina inca* | Tortolita |  |
|  |  | *Columbina passerina* | Torito | S |
|  |  | *Zenaida asiatica* | Paloma tehuacanera  Torcasa |  |
|  |  | *Zenaida macroura* | Lloronsita | **S** |
| Cuculiformes | Cuculidae | *Piaya cayana* | SN |  |
|  |  | *Geococcyx velox* | Correcaminos |  |
| Caprimulgiformes | Caprimulgidae | *Nyctidromus albicollis* | Tapacaminos | BH |
|  |  | *Caprimulgus ridgwayi* | Tapacaminos | BH |
| Apodiformes | Trochilidae | *Cynanthus sordidus* | Chupamirto  Chuparosas | FH |
|  |  | *Cynanthus latirostris* | Chupamirto  Chuparosas | FH |
| Cathartiformes | Cathartidae | *Coragyps atratus* | Zopilote |  |
|  |  | *Cathartes aura* | Zopilote |  |
| Accipitriformes | Accipitridae | *Parabuteo unicinctus* | Águila negra | PC |
|  |  | *Buteo jamaicensis* | Águila cola roja | PC |
| Strigiformes | Tytonidae | *Tyto alba* | Lechuza |  |
|  | Strigidae | *Glaucidium brasilianum* | Tecolotito |  |
|  |  | *Micrathene whitneyi* | Tecolotito |  |
|  |  | *Aegolius acadicus* | Totopito con chilaquil | S |
| Trogoniformes | Trogonidae | *Trogon elegans* | Pájaro coa  Pájaro bandera | PC |
| Coraciiformes | Momotidae | *Momotus mexicanus* | Pájaro reloj | BH  M |
| Piciformes | Picidae | *Melanerpes hypopolius* | Pájaro carpintero |  |
|  |  | *Picoides scalaris* | Pájaro carpintero |  |
| Falconiformes | Falconidae | *Falco sparverius* | Gavilán pollero | FH  D |
|  |  | *Falco peregrinus* | Gavilán |  |
| Passeriformes | Tyrannidae | *Camptostoma imberbe* | Mosquerito | FH |
|  |  | *Pyrocephalus rubinus* | Rayito  Pájaro del rayo  San Gabrielito | PC  RH |
|  |  | *Myiarchus tuberculifer* | Bolillero | FH |
|  |  | *Tyrannus melancholichus* | Injambreros | FH |
|  | Laniidae | *Lanius ludovicianus* | Chape brujo | FH |
|  | Corvidae | *Aphelocoma californica* | Cutavia |  |
|  |  | *Corvus corax* | Cacalote |  |
|  | Hirundinidae | *Stelgidopteryx serripennis* | Golondrina |  |
|  |  | *Hirundo rustica* | Golondrina |  |
|  | Troglodytidae | *Catherpes mexicanus* | Saltapared | BH  M |
|  |  | *Troglodytes aedon* | Saltapared | BH  M |
|  |  | *Thryomanes bewickii* | Saltapared | BH  M |
|  |  | *Campylorhynchus jocosus* | Pishishe | S |
|  |  | *Campylorhynchus brunneicapillus* | Pishishe | S |
|  | Polioptilidae | *Polioptila caerulea* | Perlita |  |
|  |  | *Polioptila albiloris* | Perlita |  |
|  | Mimidae | *Toxostoma curvirostre* | Cuicuite | S |
|  |  | *Mimus polyglottos* | Chape | S  BH |
|  | Ptiliogonatidae | *Phainopepla nitens* | Copetón  Ruiseñor | PC  FS |
|  | Fringillidae | *Haemorhous mexicanus* | Gorrión rojo | PC |
|  |  | *Spinus psaltria* | Dominico |  |
|  | Passerellidae | *Aimophila ruficeps* | Chillón | S |
|  |  | *Aimophila notosticta* | Chillón | S |
|  |  | *Peucaea humeralis* | Chillón | S |
|  |  | *Peucaea mystacalis* | Chillón | S |
|  | Icteridae | *Quiscalus mexicanus* | Urraca |  |
|  |  | *Molothrus aeneus* | Gañan | RH |
|  |  | *Icterus wagleri* | Calandria |  |
|  |  | *Icterus cucullatus* | Calandria fina  Calandria española | PC |
|  |  | *Icterus pustulatus* | Calandria amarilla |  |
|  | Parulidae | *Oreothlypis celata* | Chipe |  |
|  | Cardinalidae | *Pheucticus chrysopeplus* | Bionche |  |
|  |  | *Pheucticus melanocephalus* | Bionche |  |
|  |  | *Cyanocompsa parellina* | Gorrión azul | PC |
|  |  | *Passerina caerulea* | Gorrión Azul | PC |
|  |  | *Passerina cyanea* | Azulejo | PC |
|  | Passeridae | *Passer domesticus* | Chillón | S |

The local name is associated with:

**S:** song; **PC:** plumage color; **FH:** feeding habits; **RH:** reproductive habits; **BH:** behavioral habits; **FS:** feather shape: **M:** movements; **D:** damage to the population.
